# Management of Skin Toxicities in Cancer Treatment: An Australian/New Zealand Perspective

**DOI:** 10.3390/cancers16142526

**Published:** 2024-07-12

**Authors:** Rahul Ladwa, Gerald Fogarty, Peggy Chen, Gurpreet Grewal, Chris McCormack, Victoria Mar, Delphine Kerob, Kiarash Khosrotehrani

**Affiliations:** 1 Princess Alexandra Hospital, Ipswich Road, Woolloongabba, QLD 4102, Australia; 2 Faculty of Medicine, University of Queensland, Herston, QLD 4006, Australia; 3 Icon Cancer Centre Revesby, Revesby, NSW 2212, Australia; 4 Peggy Chen Skin Cancer and Mohs Surgery, New Plymouth 4310, New Zealand; 5 Te Whatu Ora Health New Zealand Taranaki, Westtown, New Plymouth 4310, New Zealand; 6 McGrath Foundation Breast Care Nurse, Alfred Health, Cancer Services, Melbourne, VIC 3127, Australia; 7 Department Surgical Oncology, Peter MacCallum Cancer Centre, Melbourne, VIC 3052, Australia; 8 Victorian Melanoma Service, Alfred Health, Melbourne, VIC 3004, Australia; 9 School of Public Health and Preventive Medicine, Monash University, Melbourne, VIC 3800, Australia; 10 La Roche-Posay International, 92300 Levallois, France; 11 Dermatology Research Centre, Experimental Dermatology Group, Frazer Institute, The University of Queensland, Woolloongabba, QLD 4072, Australia; 12 Department of Dermatology, Princess Alexandra Hospital, Woolloongabba, QLD 4102, Australia

**Keywords:** dermatological toxicities, cancer, skin toxicity, chemotherapy, radiotherapy, Australia, New Zealand

## Abstract

**Simple Summary:**

Many cancer treatments, including chemotherapy, targeted therapy, immunotherapy, and radiotherapy, can cause skin side effects. These are called ‘dermatologic toxicities’ or ‘skin toxicities’. There are many different types of skin toxicities, some of which can not only affect the quality of life but also lead to cancer treatment being stopped or slowed down. This paper gives an overview of 12 of the most common skin toxicities experienced by people receiving cancer treatment. These include rashes, dry skin, skin irritation, hair loss, changes in skin colouring, and itching. We have provided Australia/New Zealand-specific recommendations on how skin toxicities can be prevented and managed, including the role of dermocosmetic solutions.

**Abstract:**

Cancer systemic therapeutics and radiotherapy are often associated with dermatological toxicities that may reduce patients’ quality of life and impact their course of cancer treatment. These toxicities cover a wide range of conditions that can be complex to manage with increasing severity. This review provides details on twelve common dermatological toxicities encountered during cancer treatment and offers measures for their prevention and management, particularly in the Australian/New Zealand context where skincare requirements may differ to other regions due to higher cumulative sun damage caused by high ambient ultraviolet (UV) light exposure. Given the frequency of these dermatological toxicities, a proactive phase is envisaged where patients can actively try to prevent skin toxicities.

## 1. Introduction

The prevalence of cancer in Australia and New Zealand is remarkably high. Australia and New Zealand have the highest age-standardised cancer incidence (including non-melanoma skin cancer except basal cell carcinoma) globally [1]. An estimated 162,162 new cancer cases were diagnosed in Australia in 2022, and 27,072 cases were diagnosed in New Zealand in 2020 [2,3]. Approximately two in five people (or 43%) will be diagnosed with cancer by the age of 85 [2]. Most patients will receive surgery, and some will then go on to have other forms of systemic treatment such as radiotherapy, chemotherapy, and targeted therapies. All systemic treatments are associated with skin changes which are commonly referred to as dermatologic toxicities [4].

Dermatological toxicities cover a range of conditions including dry skin, skin irritation, photosensitivity, pigmentation changes, and pruritus. Their diagnosis and management are noteworthy as both patients and doctors/nurses report negative effects on patients’ physical, functional, emotional, and social well-being [5]. In severe cases, dermatological toxicities can lead to poor treatment adherence and drug cessation, altering treatment response and overall survival [4,6]. A longitudinal prospective study of cancer patients also found that 65.8% of patients developed grade 1 or higher skin toxicity within 3 months of cancer therapy [7]. Similarly, a single-centre, cross-sectional observational study reported that 56% of patients receiving antineoplastic drugs developed at least one dermatological toxicity [8]. Therefore, it becomes logical for patients receiving anti-cancer therapy to receive proper information regarding the cutaneous adverse events that may occur and initiate prophylactic skincare management.

Rates of different dermatological toxicities vary depending on the treatment type and duration. One of the most common toxicities is reported as a rash [9]. Some anti-cancer therapeutics are particularly problematic to the skin. For example, most patients who receive epidermal growth factor receptor inhibitor (EGFRi) in the treatment of metastatic colorectal cancer develop skin side effects, with approximately 10% to 20% of patients experiencing grade 3/4 toxicity [10]. In 2007, 32% of oncology providers reported discontinuing EGFRi therapy due to a rash [11]. Hair loss, hyperpigmentation, and dry skin are also frequently reported skin toxicities [7].

Skincare requirements in Australia and New Zealand may differ from other regions due to pre-existing sun damage caused by increased ambient ultraviolet (UV) light and high cumulative sun damage due to an outdoor lifestyle. Despite both countries being in the southern hemisphere, Australia has higher maximum UV levels as many of its regions are closer to the equator; it also has a greater immigrant population from southern Europe with fair skin [12]. This is reflected in rates of skin cancer, with more than two in three Australians expected to develop at least one keratinocyte cancer in their lifetime [13]. Moreover, the availability of dermoscosmetics and various anticancer medications in this region may also differ from other countries. As such, specific consideration may be needed for this region. In 2023, an expert consensus statement entitled “The role of dermocosmetics in the management of cancer-related skin toxicities” was developed by an international team of dermatology and oncology specialists [14]. The aim of the current publication is to develop Australia/New Zealand-specific recommendations based on the international consensus statement recommendations [14]. An advisory board was held in May 2023 with the authors to review these recommendations in the Australian/New Zealand context, and these are discussed below.

In principle, many aspects of the management strategy rely on adequate information about patients undergoing the therapy as well as the oncology treating team. Given the frequency of these side effects, a proactive phase is envisaged where the patients can actively try to prevent skin toxicities. However, if the anti-cancer therapy skin side effects are apparent, this guide provides a separate set of recommendations for their management.

## 2. Acute Radiation Dermatitis

Acute radiation dermatitis is a cutaneous reaction caused by ionising radiation [15]. It typically occurs gradually as radiation is delivered, and for up to 90 days post-delivery [16]. Reaction severity can range from mild erythema to moist desquamation and ulceration [15]. Reaction severity can be enhanced by concomitant treatment with chemotherapy or targeted therapy (particularly 5-fluorouracil and EGFRi).

### 2.1. Prevention

Current measures employed to prevent these acute reactions are proper skin hygiene and topical steroids [15]. To prevent treatment interruption and infection, there is a critical need for patient education by nurses, follow-up, and the initiation of early management strategies. Prophylactic measures recommended for acute radiation dermatitis include skincare with gentle cleansers and moisturisers that need to be initiated at the start of radiation therapy (Table 1). In the proactive phase, a daily moisturiser can be used from the start of treatment to replace the natural moisture that is no longer being produced by the skin appendages. To avoid influencing the radiotherapy treatment, moisturisers should not contain any metals nor be applied within 2 h before radiation therapy.

The use of mild potency topical corticosteroids (TCSs) (particularly betamethasone) may be used if the skin is not the target of radiotherapy (i.e., where the skin is being inadvertently irradiated) as it can improve discomfort and itching. However, TCS should be avoided if the skin is the target of radiotherapy as steroids may interfere with the radiation response [17]. Preventative skincare for acute radiation dermatitis is only effective if the patient is well-nourished. As such, patients should be weighed weekly to monitor their nutritional status.

### 2.2. Management

Gentle skincare is recommended for the management of acute radiation dermatitis (Table 1). When moist desquamation and superficial erosions occur, moisturisers are no longer used and are replaced by a gel dressing. This accelerates the migration of normal new skin cells across the denuded dermis. Dressing may be required, and specialist nursing care is advised. The use of lidocaine 2% gel for pain management, however, it is not commonly used in Australia and New Zealand: a cold compress is used for pain management. The use of powder for moisture removal is not advisable as it can delay skin healing and cause fibrotic reactions.

It is important for patients to avoid irritants such as hot showers, sunlight, and trauma to the skin during skin radiotherapy. When applying dressings to the skin, the extent of the radiotherapy field on the skin is important to know so that dressing adhesive is not applied to the field as this would constitute trauma.

Secondary infections of acute radiation dermatitis must be monitored. Systemic or topical antibiotics (as advised by the oncologist/dermatologist) can be used in the case of a secondary infection. In the case of grade 3 ulceration, radiation oncologists may choose to interrupt treatment.

## 3. Chronic Radiation Dermatitis

Chronic radiation dermatitis is a late side effect of skin radiation. Systemic therapies, radiotherapy techniques, the individual’s genetic, and health factors, as well as phenotype, phototype, and blood supply to the treated volume, can predispose some patients to this condition [18]. Chronic radiation dermatitis can lead to skin alterations such as necrosis, fibrosis, and ulceration [18]. More severe but rarer side effects include the development of a fistula (an abnormal connection between an organ and the skin), radiation-induced angiosarcomas, and radiation-induced second malignancy [19,20]. Another rare, late side effect of skin radiotherapy is radiation recall [19,21], whereby an inflammatory reaction occurs when certain anticancer drugs are given after radiation therapy. Radiation recall usually affects the part of the body that received radiation, especially the skin.

### 3.1. Prevention

The most effective measure to avoid chronic radiation dermatitis is the implementation of proper radiation therapy techniques so that healthy skin is not irradiated (Table 1). Gentle cleansers, moisturisers, and sun protection are also recommended.

### 3.2. Management

Currently, the available evidence on the management of chronic radiation dermatitis is lacking. Therapies include a trial of pentoxifylline and vitamin E [22], as well as hyperbaric oxygen therapy [23] (Table 1).

## 4. Alopecia from Chemotherapy

Chemotherapy-induced alopecia describes the loss of head and body hair in response to certain chemotherapy drugs such as alkylating agents, antimetabolites, and mitotic inhibitors. With an estimated incidence of 65%, the severity of hair loss is dependent on the drug type and dose, as well as patient-related characteristics such as comorbidities and hormonal status [24]. This kind of alopecia is usually reversible however hair colour and texture can change following regrowth.

### 4.1. Prevention

Currently, no guidelines exist for the management of chemotherapy-induced alopecia [24]. Prophylactic measures include the use of soft hairbrushes, gentle shampoos, and moisturising creams for gentle haircare (Table 1). Scalp cooling and cold caps can also be used if available (Table 1); scalp cooling is believed to promote vasoconstriction, potentially reducing the drug dose that reaches the hair follicle [24]. A meta-analysis of 1098 participants undergoing chemotherapy found that scalp cooling significantly reduced the risk of chemotherapy-induced alopecia (RR = 0.38, 95% CI = 0.32–0.45), with no serious adverse effects [25].

### 4.2. Management

There are no proven pharmaceutical products for the management of this condition. Cosmetic approaches such as microblading/semi-permanent tattooing of eyebrows may be used to boost the patient’s self-esteem (Table 1). However, these procedures can introduce the risk of infection in chemotherapy patients and should be approved by the oncologist. Topical bimatoprost can be considered with eyelash loss as it enhances eyelash growth, length, and thickness (Table 1); however, bimatoprost has not been approved for this indication. Post-chemotherapy management of chemotherapy-induced alopecia can include the use of topical minoxidil and antioxidants to promote hair regrowth after a reasonable time lapse from the end of therapy [24].

## 5. Alopecia from Hormonal and Targeted Therapies

Hormones have a significant impact on hair growth. As such, endocrine therapies such as aromatase inhibitors and anti-estrogens can have a significant impact on hair growth and loss [26]. The overall incidence of alopecia from hormone therapy is 4.4%; however, the highest incidence (25.4%) is observed in patients treated with tamoxifen [27]. Targeted anticancer therapies can also cause alopecia, with an overall incidence of 14.7% [28]. Although alopecia is not dose-limiting or life-threatening, it can greatly impact patients’ quality of life and well-being.

### 5.1. Prevention

Prophylactic measures include the use of gentle shampoos and moisturising creams for gentle haircare (Table 1).

### 5.2. Management

The role of micronutrients in alopecia has been studied; however, there is not enough evidence to support their use [29]. Studies have shown a relationship between alopecia and low vitamin D levels, and the correction of vitamin D deficiency can improve outcomes [29]. As such, oral vitamins are recommended to help promote hair regrowth (Table 1). Minoxidil 5% is also a treatment option (Table 1) as it has been shown to promote hair growth and has become a mainstay treatment for alopecia [30]. Anti-androgenic drugs such as spironolactone are used to treat androgenetic alopecia. Spironolactone exhibits better efficacy in combination with other therapies such as oral or topical minoxidil compared with monotherapy [31].

## 6. Hypertrichosis and Trichomegaly

Hypertrichosis (the development of small vellus hairs on the female chin and lip) and trichomegaly (the development of long, rigid, curly eyelashes) are common cutaneous side effects observed in EGFRi-treated patients [32,33]. Approximately 62% of patients treated with EGFRIs for longer than 6 months exhibit eyelash trichomegaly, and 56% exhibit hypertrichosis [34].

### 6.1. Prevention

There are no prophylactic treatments for these two conditions.

### 6.2. Management

Management of trichomegaly can include eyelash clipping, and patients can be referred to an ophthalmologist if eye irritation occurs (Table 1). Patients with hypertrichosis can choose cosmetic hair removal techniques including laser, epilation, depilatory creams, and threading (Table 1).

## 7. Xerosis/Pruritus

Xerosis and pruritus are common side effects of many cancer therapies. Pruritus relates to an itch caused by the administration of a drug [35], commonly associated with targeted cancer therapies. The overall incidence of pruritus in patients treated with targeted cancer therapies has been reported at 17.4% [36]. Pruritus is also caused by cytotoxic chemotherapy, radiation therapy, and immune checkpoint inhibitors [37]. Xerosis (dry skin) is common in patients receiving many types of chemotherapy and radiation, including EGFRi. Xerosis is most pronounced on the extremities and can progress to acral fissuring and asteatotic eczema [38]. Many targeted cancer therapies can lead to xerosis, with an overall incidence of 17.9% [39].

### 7.1. Prevention

Pruritus and xerosis can be avoided by initiating preventative measures at the same time as cancer therapy. Daily application of moisturisers with key ingredients such as shea butter, niacinamide, and ceramides [40] is recommended (Table 1). Moisturisers should be applied to the face, hands, feet, lower legs, and neck as these are common sites of pruritus development. For washing, gentle cleansers close to skin pH with lipid-replenishing ingredients should be used (Table 1).

### 7.2. Management

Continuation of the daily application of moisturisers with key ingredients such as shea butter, niacinamide, and ceramides is recommended, as well as the use of hydrating lip balms (preferably containing sunscreen). In the case of severe xerosis, balms and creams containing 3–10% urea should be used. Urea is used in dermatology due to its excellent hydrating and moisturising properties and has been shown to improve pruritus [41]. If there is an eczematous eruption, TCS or topical calcineurin inhibitors (TCIs) can be used one to two times per day. In the case of pruritus, topical preparations with menthol (0.5–2%) or camphor (0.5%), antihistamines or doxepin/aprepitant/antidepressants, and transcutaneous electrical nerve stimulation (TENS) or acupuncture can also be used (Table 1). Antihistamines that bind to the histamine 1 receptor (H1) serve as important therapeutic agents to counter the effects of histamine in the skin and have been shown to greatly reduce histaminergic pruritus [42]. However, anti-H1 does not work well on non-histaminergic pruritus. Oral antidepressants such as doxepin may treat pruritus and could be used in patients unresponsive to topical treatment and oral antihistamines [43]. Neurostimulation via TENS may ameliorate pruritus itching and can be an inexpensive treatment for patients with a refractory chronic itch [44,45]. Acupuncture can also alleviate itching and may be used in patients with pruritus; however, it poses a risk of infection in patients undergoing systemic therapy. A meta-analysis of three RCTs found that acupuncture therapy was effective in alleviating itching compared with placebo acupuncture or no treatment group [46]. UV therapy, particularly narrowband UVB therapy, may also be helpful for pruritus if not contraindicated.

## 8. Drug-Induced Maculopapular Rash

A maculopapular rash is the most common type of skin reaction to anti-cancer drugs [47] and is the most frequent adverse event reported with checkpoint inhibitors [48]. These rashes can significantly impact patients’ physical and emotional well-being.

### 8.1. Prevention

The application of moisturisers/emollients/balms with key ingredients such as shea butter, niacinamide, and ceramides is recommended (Table 1). Sun protection using sunscreen with UV-broad spectrum UVA/UVB filters is advised in order to minimise possible post-inflammatory hyperpigmentation (PIH) in darker phototype patients.

### 8.2. Management

For mild drug-induced maculopapular rash, the continued application of moisturisers as described above is recommended. A topical antipruritic cream, including multi-formulation camphor–menthol–hydrocortisone cream, is recommended to relieve itching and pain (Table 1). Camphor exhibits analgesic and anti-inflammatory properties and promotes blood flow in the skin [49]. Menthol at a concentration of 1–3% promotes skin cooling, which has been shown to provide therapeutic relief from itching [50]. For a more moderate maculopapular rash, TCS (once–twice per day for a maximum of 4 weeks) and oral antihistamines can be used to relieve maculopapular rash [51]. The location of the rash (e.g., trunk versus face), severity, and extent will determine the potency of the topical steroid used. In general, creams and lotions are more easily applied to hair-bearing skin while ointments are thicker but will also act as an emollient and will penetrate skin better. Oral corticosteroids can be also considered for grade 3 toxicities. Severe (grade 4) maculopapular rash requires hospital admission (Table 1).

## 9. Acneiform Rash/Folliculitis

Acneiform rash (papulopustular eruption) is characterised by an eruption of papules and pustules on the face, scalp, upper chest, and back [35]. It is one of the most frequently reported adverse events induced by targeted therapies, particularly EGFRi [35].

### 9.1. Prevention

To prevent rash development, preventative agents such as gentle cleansers and moisturisers as well as oral doxycycline (100 mg BID) or minocycline are recommended upon commencement of EGFR and MEK inhibitor therapies (Table 1).

### 9.2. Management

Following rash development, barrier repair and moisturising topical agents (including acne dermocosmetics where appropriate) are recommended (Table 1). For rash progression, minocycline, doxycycline, topical corticosteroids, or systemic steroids should be used. Referral to a dermatologist should take place where there is no response to increasing doses of these agents and early referral is encouraged where possible. Ideally, dermatologists should be introduced to the oncology multidisciplinary team to manage these oncotherapy-induced rashes.

Following acneiform rash development, moisturising creams/balms, barrier repair creams (with ingredients such as panthenol for microbiome rebalancing), and acne dermocosmetics with nicotinamide are recommended (Table 1). The pharmaceuticals minocycline/doxycycline, systemic steroids, and TCS (where there is no local superinfection) are also recommended for acneiform rash (Table 1). Ultimately, dose adjustment of EGFR inhibitors may help overcome a difficult acneiform eruption.

More recently, EGF ointments have been proposed for treating EGFR inhibitor-induced skin toxicities, including acneiform rash [52,53]. A randomised controlled phase III trial of 80 cancer patients treated with EGFR inhibitors showed that the application of EGF ointment twice daily significantly improved skin toxicities (77.8% response rate using 20 ppm EGF concentration) and patient quality of life [52]. Similarly, an open-label, non-comparative, multicentre, phase II trial of 46 cancer patients treated with the EGFR inhibitor erlotinib showed that EGF ointment was effective in treating 69.2% of patients [53]. These promising results await application in the clinic.

## 10. Photosensitivity

Skin photosensitivity refers to a range of skin conditions that are induced or exacerbated by exposure to the electromagnetic spectrum of sunlight [54]. Such skin conditions include the typical exaggerated sunburn response, photo-distributed lichenoid dermatitis, hyperpigmentation, and autoimmune conditions such as lupus potentially manifesting as annular lesions [54]. Photosensitivity in anti-cancer medications is often through direct toxicity and is induced mainly through UVA [55] and caused by many topical or systemic anti-cancer therapies including BRAF inhibitors (particularly vemurafenib) [56], multikinase inhibitors, vandetanib, chemotherapy (fluorouracil, dacarbazine, methotrexate, paclitaxel, vinblastine), and radiation therapy. Patients with darker phototypes are at higher risk for PIH.

### 10.1. Prevention

Oncology patients should be advised to avoid direct UVA and UVB exposure, especially at midday when the UV index is greatest. Sunscreen with broad spectrum protection, including a high UVA PF, should be reapplied every two hours, and tinted sunscreens should be used for further visible spectrum sun protection. Patients should also be encouraged to take protective measures such as the thorough application of UV-broad spectrum UVB/UVA SPF 50 or greater sunscreens and facilitate skin coverage with clothing, sunglasses, and hats. They should be encouraged to stay in the shade as much as possible. Patients should also be educated on potential sun exposure through glass windows and in cloudy weather (Table 1).

### 10.2. Management

In addition to the prophylactic measures listed above, patients can be advised to use cooling agents such as aloe vera, soothing emollients, and cool colloidal oatmeal baths. For more severe photosensitivity, non-steroidal, anti-inflammatory drugs (NSAIDs) can be used for their anti-inflammatory effect and pain relief if there are no contraindications. Other over-the-counter analgesics can be recommended for pain relief. Topical corticosteroids can also be used for acute cases of photosensitivity (Table 1).

## 11. Skin/Nail Pigmentation Changes

A wide range of cancer therapeutics can cause nail changes such as melanonychia, leukonychia, Beau’s lines, and onychomadesis [57]. These changes are often cosmetic and resolve after treatment cessation. However, some changes can be painful and affect patients’ quality of life.

### 11.1. Prevention

Measures to reduce the risk of skin/nail pigmentation changes include the use of a gentle cleanser with pH ~ 5 and the avoidance of sunlight, heat, and humidity. Sunscreen should be reapplied every two hours. Patients should be educated to wear broad-spectrum UVB/UVA SPF 50 or greater sunscreens as well as protective clothing (Table 1). Opaque nail lacquers can be used for nail protection from visible spectrum sun radiation.

### 11.2. Management

In addition to the prophylactic measures listed above, patients should be advised to use topical preparations containing niacinamide as this has been shown to reduce cutaneous hyperpigmentation by inhibiting melanosome transfer from melanocytes to keratinocytes [58]. For more severe pigmentation changes, TCS can be used. Hydroquinone is a skin-lightening agent; skin-lightening agents such as hydroquinone should not be used within 6 months of anti-cancer therapy cessation, and the treatment duration should not exceed 6 months.

## 12. Inflammatory Hand–Foot Syndrome

Hand–foot syndrome (also called palmar–plantar erythrodysaesthesia syndrome (PPES), acral erythema, or toxic erythema) is a common complication of many targeted chemotherapeutics (including liposomal doxorubicin and mitotic inhibitors) and capecitabine [59]. Hand–foot syndrome starts as redness, swelling pain, and tingling in the palms of the hands or soles of the feet [35,38]. In a study of skin toxicities caused by chemotherapy, hand–foot syndrome was a stronger factor in decreasing quality of life than xerosis, paronychia, pigmentation, or rash [60]. Currently, the most effective management for this condition is treatment interruption or dose intensity modification, with symptoms generally resolving within 1–3 weeks of chemotherapy cessation [59].

### 12.1. Prevention

Prophylactic measures can be taken to prevent hand–foot syndrome, including the topical use of 10% urea-based moisturisers (Table 1), as urea appears to be effective in the prevention of hand–foot syndrome [61]. Creams containing 6% salicylic acid may also be recommended [62]. Local cooling has also been shown to alleviate hand–foot syndrome pain and reduce the frequency and severity of the condition [63]; prophylactic use of cooling gloves can help; however, this technique is not widely used in Australia and New Zealand since the required facilities are not readily available. Oral steroids and the COX-2 inhibitor celecoxib can also be considered (Table 1); however, supporting evidence for the efficacy of these measures is lacking due to a paucity in randomised controlled trials [64].

### 12.2. Management

Treating hand–foot syndrome involves the continuation of prophylactic measures. Depending on the severity of the condition, TCS, oral dexamethasone, and the interruption of capecitabine can be used (Table 1). If there is no response to these treatments, cancer therapy interruption or dose modification may be considered by the oncologist.

## 13. Hyperkeratotic Hand–Foot Syndrome

Hyperkeratotic hand–foot syndrome, occurring in approximately 30% of patients, is a skin reaction to some multikinase and RAS-BRAF-MEK-ERK inhibitors [65]. This condition is histologically different to inflammatory hand–foot syndrome and is characterised by yellowish hyperkeratotic plaques which often present with tingling, burning, and numbness sensations [65].

### 13.1. Prevention

Prophylactic measures for this condition include the use of gentle cleansers (pH ~ 5), 10% urea moisturisers, and avoidance of high pressure or high friction on the feet by wearing soft footwear (Table 1). A potent TCS such as clobetasol propionate may also be initiated [66].

### 13.2. Management

In addition to the continuation of the prophylactic measures, hyperkeratotic hand–foot syndrome should be managed using a topical 10–40% urea-based emollient to aid natural exfoliation and referral to a podiatrist (Table 1) [66]. On a practical aspect, plantar fissures need to be covered by barrier cream such as zinc paste. For pain management, a topical analgesic such as lidocaine 2% can be used for localised relief [66]. Treatment can also include systemic analgesics or NSAIDs and corticosteroids as needed (Table 1) [66,67]. Finally, patients need to be monitored for infection using gentle antiseptics to prevent the expansion of weeping areas.

## 14. Practical Considerations in the Management of Oncotherapy-Induced Skin Toxicities

### 14.1. Sun Protection

Due to higher maximum UV levels, Australia and New Zealand have the highest rates of cutaneous malignant melanoma globally [12]. As such, given the increased susceptibility of people undergoing cancer therapy to cutaneous sun damage and melanoma, patients living in Australia and New Zealand must exercise stringent sun protection measures. Sun protection including sunscreen for non-covered areas should be strongly recommended to follow moisturisers for proactive prevention of side effects.

### 14.2. Patient Education

Where and when patients are given skincare education is critical. Patients should not be overburdened with skincare education at initial oncology visits. Nurses can take on this education role at later visits; skincare education can be provided at the time patients receive formal pretreatment education. Since cancer therapies can affect skin differently, the advice given should be tailored to the patient’s specific treatment. Nurses should re-visit skincare advice throughout the patient’s journey and when skin adverse events arise. As such, better dermatological education should be provided to nurses on how to manage mild/early skin adverse effects and when to request a medical review or refer patients to a dermatologist. Moreover, better dermatological education should be provided to pharmacists and registrars training in oncology.

### 14.3. Access to Dermatologists

There is a need for greater integrated care across oncology and dermatology in order to reduce the impact of commonly occurring dermatological toxicities and improve patient outcomes [68]. In the case of the most common dermatological side effect, rash, oncology therapies are often discontinued without dermatological consultation [9]. Early involvement of a dermatologist in the treatment of cutaneous adverse events has been shown to decrease treatment interruption and improve quality of life [69]. However, an estimated 84% of cancer patients who develop dermatological issues are not referred to a dermatologist [70]. Clear guidelines should be given to the oncology team regarding when to make a dermatological referral. These guidelines should outline what constitutes the mild/moderate/severe classifications of adverse events and at which stage of severity to refer to a dermatologist.

### 14.4. Radiation Therapy

Simple moisturisers should be used from the beginning of treatment as the organs that supply natural skin moisture, the hair follicles and sweat glands, are quickly destroyed by radiation therapy. However, metal-based creams should be avoided within the radiation area during therapy as this may perturb the radiation beam. Gel dressings are beneficial in the later stages of skin radiotherapy to accelerate normal cell migration and closing of broken skin. These may need to be wet dressings. TCS should not be used when the skin is the target of radiation therapy; however, it may be used when the skin is a bystander. Trauma and direct sun to areas being treated should be avoided as these will aggravate acute toxicity.

## 15. Conclusions

Skin toxicity is common and can negatively impact the biopsychosocial well-being of patients with a cancer diagnosis and can eventually determine the discontinuation of therapy. Education in the prevention of skin toxicities can improve outcomes for patients. Prompt awareness of skin toxicities and their treatment in conjunction with oncodermatology input has the potential to maximise effective cancer treatment whilst limiting severe toxicities. Specialised nursing input and follow-up are vital in providing patients with adequate education and the early identification and management of skin toxicities.

## Figures and Tables

**Table 1:** Recommendations for the management of skin toxicities in Australia and New Zealand. Modified from the international expert consensus [14].

Type of Treatment	Commonly Used Therapeutics	Proactive Phase (Prevention of AE)	Reactive Phase (Management of AE)
**Acute radiation dermatitis**			
Radiation therapy, enhanced by concomitant therapy with chemotherapy or targeted therapy (especially 5-fluorouracil and EGFR inhibitors), and sometimes by checkpoint inhibitors	Cetuximab, erlotinib, gefitinib	Gentle soap-free wash with pH ~ 5 Daily moisturiser but not to be applied within 2 h before radiation therapy Sun protection when outside with UV-broad spectrum UVB/UVA SPF 50 or greater sunscreens and strong UVA protection, especially with targeted therapy; clothing, sunglasses, and hats; patients with darker phototypes are at higher risk for PIH	Gentle skincare
			Mild potency TCS (recommended only when the skin is not the target of the radiotherapy) Monitor for secondary infection Antibiotics (as directed by the oncologist/dermatologist) in the case of superinfection Radiation oncologist may interrupt treatment in case of grade 3 ulceration.
**Chronic radiation dermatitis**			
Radiation therapy, enhanced by concomitant therapy with chemotherapy or targeted therapy (especially 5-fluorouracil and EGFR-I), and sometimes by checkpoint inhibitors	Radiotherapy	Gentle soap-free wash with pH ~ 5 Moisturiser Sun protection	Gentle skincare Educate patient about risk of late development of skin cancers in treated areas
			Pentoxifylline 400 mg TID to prevent radiation-induced fibrosis (in the latter part of radiation therapy or following the development of fibrosis; use for at least 6 months as long as it is still improving the condition) Oral vitamin E (use with caution in patients taking anticoagulant or antiplatelet therapy)
**Alopecia from chemotherapy**			
Conventional chemotherapy (alkylating agents, antimetabolites, mitotic inhibitors)	Taxanes: Docetaxel Paclitaxel Ciclophosphamide, Ifosamide Vincristine, cisplatin, carboplatin, doxorubicin, donaurubicin	Moisturising cream Gentle shampoo Scalp cooling and cold caps for cytotoxic infused chemotherapy, if available (Contraindications: hematologic cancers and cancers of head/neck, those receiving platinum-based therapy due to cold sensitivity)	Microblading/semi-permanent tattooing of eyebrows (this poses a risk of infection to immunocompromised patients and would need an oncologist’s prior approval).
			Topical bimatoprost can be considered for eyelash loss
**Alopecia from hormonal and targeted therapies**			
Endocrine therapy Kinase inhibitors	Aromatase inhibitors Tamoxifen Vismodegib, sonidegib	Gentle shampoo Moisturising cream	Oral vitamins (consider zinc in zinc-deficient patients)
			Topical 5% and oral minoxidil Anti-androgen therapy such as spironolactone (for hormonal therapy) if there are no contraindications
**Hypertrichosis and trichomegaly**			
EGFR inhibitors	Cetuximab, erlotinib	None	Hypertrichosis: cosmetic techniques, various epilation techniques, threading Trichomegaly: eyelash clipping, referral to ophthalmologist
**Xerosis/pruritus**			
Targeted therapy (kinase inhibitors) Cytotoxic chemotherapy Radiation therapy Immune checkpoint inhibitors	Vemurafenib, dabrafenib Cyclophosphamide Nivolumab, pembrolizumab	Initiate at the same time as cancer treatment Gentle soap-free wash close to skin pH with lipid-replenishing ingredient Moisturisers, including moisturisers able to maintain diverse skin microbiomes (emollient “plus”), with key ingredients such as shea butter, niacinamide, and ceramides Apply moisturiser to face, hands, feet, neck, and back daily plus re-apply as needed	Gentle cleanser close to skin pH with lipid-replenishing ingredient Emollients “plus” balm, able to rebalance microbiome, with key ingredients such as shea butter, niacinamide, and ceramides Preferred formulas: Moisturising cream and balms Urea 3–10% in case of severe xerosis avoid in red/irritated areas and with radiation dermatitis Hydrating lip balms, preferably with sunscreen Topical antipruritics: Camphor-menthol-cream
			If eczematiform eruption: TCS/TCI once–twice per day (maximum of 4 weeks for TCS) might be needed if pruritus uncontrolled by DC Antihistamines Doxepin/aprepitant/antidepressants TENS or acupuncture
**Drug-induced maculopapular rash ^1^**			
Targeted therapy (kinase inhibitors) Conventional chemotherapy ○ Antimetabolites (5-FU etc.) ○ Alkylating agents (cyclophosamide, cisplatin) ○ Mitotic inhibitors (taxanes, vinca alkaloids) Immunotherapy (immune checkpoint inhibitors)	Vemurafenib, dabrafenib Capecitabine, 5fluorouracil cyclophosamide, cisplatin docetaxel, vincristine pembrolizumab, nivolumab	Emollients “plus” balm, able to rebalance microbiome, with key ingredients such as shea butter, niacinamide, and ceramides Moisturisers with key ingredients such as shea butter, niacinamide, ceramides Sunscreen with UV-broad spectrum UVA/UVB filters to prevent possible PIH in darker phototypes	Continue proactive phase treatment Topical antipruritics: Camphor-menthol-hydrocortisone cream
			TCS (once–twice per day for a maximum of 4 weeks) Oral antihistamines Topical/Oral/IV corticosteroids for grade 3 toxicities Oncologist may suspend or alter treatment at grade 3 adverse events (seek dermatologist opinion); resume after 2 weeks if the rash has faded to grade 0–1. Grade 4, admit to hospital.
**Acneiform rash/folliculitis**			
Targeted therapy (kinase inhibitors)	Erlotinib, gefitinib	Gentle soap-free wash with pH ~ 5 Moisturiser Sunscreen with UV-brad spectrum UVA/UVB) on all sun-exposed areas. Patients with darker phototypes are at higher risk for PIH Acne dermocosmetics	Continue proactive phase treatment Moisturising cream and balms Barrier repair cream on face such as soothing balm, able to rebalance microbiome with ingredients such as panthenol Acne dermocosmetics with niacinamide
		Oral doxycycline ^2^ (100 mg BID) or minocycline when using EGFR and MEK inhibitors	Minocycline/doxycycline Topical corticosteroids after checking there is no local superinfection Systemic steroids *If not enough, oncologist may suspend or alter treatment.*
**Photosensitivity**			
Kinase inhibitors Vandetanib Chemotherapy Radiation therapy	vemurafenib Vandetanib fluorouracil, dacarbazine, methotrexate, paclitaxel, vinblastine	Gentle soap-free wash with pH ~ 5 Sun avoidance: Especially direct sun at mid-day * Sun protection when outside: with UV-broad spectrum UVB/UVA SPF 50 or greater sunscreens and strong UVA protection, especially with targeted therapy; clothing, sunglasses, and hats; patients with darker phototypes are at higher risk for PIH Reapply sunscreen q2h Educate patient that sun can penetrate glass windows and in cloudy weather (home, car, workplace)	Continue proactive phase treatment Cool colloidal oatmeal baths may help Soothing emollients Aloe vera
			NSAIDs Analgesics Topical corticosteroids
**Skin/nail pigmentation changes**			
Chemotherapy Antimetabolites (5-FU etc) Alkylating agents (cyclophosamide, cisplatin) Mitotic inhibitors (taxanes, vinca alkaloids) Kinase Inhibitors Checkpoint inhibitors (for vitiligo)	Vemurafenib, dabrafenib Capecitabine, 5fluorouracil cyclophosamide, cisplatin docetaxel, vincristine erlotinib, gefitinib pembrolizumab, nivolumab	Gentle soap-free wash with pH ~ 5 Sun avoidance: especially direct sun at mid-day Sun protection when outside with UV-broad spectrum UVB/UVA SPF 50 or greater sunscreens and strong UVA protection, especially with targeted therapy; clothing, sunglasses, and hats; patients with darker phototypes are at higher risk for PIH Reapply sunscreen q2h Opaque nail lacquers Avoid heat and humidity Avoid trauma or irritants, particularly for vitiligo	Continue proactive phase treatment topical preparations containing niacinamide
			Topical corticosteroids Hydroquinone for hyperpigmentation (should not be used within 6 months of oncotherapy cessation, and the treatment duration should not exceed 6 months) For vitiligo: topical steroids, topical JAK inhibitors, topical tacrolimus in addition to narrowband UVB or Excimer
**Inflammatory hand & foot syndrome**			
Conventional chemotherapy Liposomal doxorubicin Mitotic inhibitors (taxanes) Capecitabine (5-FU)		Moisturiser with urea 10%	Continue proactive phase treatment Moisturising cream and balms Monitor for changes in severity Change cancer treatment dose
		**Optional:** Oral steroids for doxorubicin HFS, celecoxib for capecitabine	TCS (once–twice per day for a maximum of 4 weeks) including under occlusion Oral dexamethasone For capecitabine, on/off regimens can help reduce HFS *Oncologists may reduce, interrupt, or discontinue treatment at higher grades.*
**Hyperkeratotic hand-foot syndrome**			
Targeted therapy (kinase inhibitors)	Sunitinib, sorafenib	Gentle soap-free wash with pH ~ 5 Moisturiser with urea 10% (2–3 times per day) ^3^ Remove hyperkeratosis before the start of treatment (pedicure) Soft/padded footwear, avoid walking barefoot, avoid high heels	Continue proactive phase treatment Topical urea-based 10–40% emollient (2–3 times per day on hyperkeratotic areas) Topical Salicylic acid 20–30% in white soft paraffin Refer to podiatrist
		Clobetasol proprionate ^4^	Lidocaine patches/cream (as needed for pain) Analgesics, NSAIDs Potent topical corticosteroids Oncologist may reduce, interrupt, or discontinue treatment at higher grades *Oncologist may reduce, interrupt, or discontinue treatment at higher grades.*

5-FU = f fluorouracil; AE = adverse event; BID = twice daily; BRAF = BRAF gene; CDK = cyclin dependent kinase; DC = discontinuation; EGFR = epidermal growth factor receptor gene; ERK = extracellular signal-regulated kinase; FGF = fibroblast growth factor; FGFR = FGF receptor; HFS = hand and foot syndrome; MEK = mitogen activated protein kinase; mTOR = mechanistic target of rapamycin; NSAIDS = nonsteroidal anti-inflammatory drugs; PI3K = phosphoinositide 3-kinase; q2h = every two hours; RAS = renin angiotensin system inhibitor; SPF = sun protection factor; TCI = topical calcineurin inhibitors; TCS = topical corticosteroids; TENS = transcutaneous electrical nerve stimulation; TID = three times daily; UV = ultraviolet; VEGFR = vascular epithelial growth factor. ^1^ Check that the rash is not a cutaneous toxicity that would necessitate drug interruption, viral infection, or relapse of previous skin condition. Avoid oral steroids for maculopapular rash >grade 3 without a dermatology consultation; ^2^ can cause photosensitivity; ^3^ may use higher percentage depending on patient response; ^4^ with multikinase inhibitor regorafenib.
